# Effectiveness of a multicomponent intervention to enhance implementation of a healthy canteen policy in Australian primary schools: a randomised controlled trial

**DOI:** 10.1186/s12966-016-0431-5

**Published:** 2016-10-07

**Authors:** Nicole Nathan, Sze Lin Yoong, Rachel Sutherland, Kathryn Reilly, Tessa Delaney, Lisa Janssen, Katie Robertson, Renee Reynolds, Li Kheng Chai, Christophe Lecathelinais, John Wiggers, Luke Wolfenden

**Affiliations:** 1 Hunter New England Population Health, Hunter New England Local Health District, Newcastle, New South Wales 2287 Australia; 2 School of Medicine and Public Health, The University of Newcastle, Newcastle, New South Wales 2308 Australia; 3 Hunter Medical Research Institute, Newcastle, New South Wales 2300 Australia

**Keywords:** Implementation, Schools, Nutrition, Policy, Canteen

## Abstract

**Background:**

The implementation of school nutrition policies, which govern the provision of food in schools, is recommended as a public health strategy to support the development of healthy dietary behaviours in school-aged children. Despite this, research internationally and in Australia indicates that few schools implement such policies. This study aims to examine whether a theoretically designed, multi-strategy intervention was effective in increasing the implementation of a healthy canteen policy in Australian primary schools.

**Methods:**

A parallel group randomised controlled trial was conducted with all government and Catholic primary schools within one region in New South Wales, Australia who had an operational canteen that provided food to primary school aged children (5–12 years) and were not currently receiving an intervention to change their canteen practices. Schools randomised to the intervention arm received a 9-month multicomponent intervention including ongoing support, provision of resources, performance monitoring and feedback, executive support and recognition. The primary outcomes were the proportion of the schools with a canteen menu that: i) did not include ‘red’ or ‘banned’ items according to the healthy canteen policy; and ii) had more than 50 % ‘green’ items. The primary outcome was assessed via menu audit at baseline and follow up by dietitians blinded to group allocation.

**Results:**

Fifty-three eligible schools were randomised to either the intervention or control group (28 intervention; 25 control). Analyses with 51 schools who returned school menus found that intervention schools were significantly more likely relative to control schools to have a menu without ‘red’ or ‘banned’ items (*RR* = 5.78 (1.45–23.05); *p* = 0.002) and have at least 50 % of menu items classified as green (*RR* = 2.03 (1.01–4.08); *p* = 0.03).

**Conclusions:**

This study found that a multi-component intervention was effective in improving primary schools’ compliance with a healthy canteen policy. Given the lack of evidence regarding how best to support schools with implementing evidence-based policies to improve child diet, this trial for the first time provides high quality evidence to practitioners and policy makers seeking to improve nutrition policy implementation in schools.

**Trial registration:**

This trial was prospectively registered with the Australian New Zealand Clinical Trials Registry (ACTRN12614001148662) 30th October 2014.

## Background

Poor dietary behaviours are associated with the development of numerous chronic diseases including cardiovascular disease [1], some cancers [2], stroke [3] and type 2 diabetes [4]. Evidence suggests that a large proportion of children in high income countries, including the United States [5, 6], United Kingdom [7], and Australia [8] do not meet national dietary guidelines [5–8]. As dietary behaviours established in childhood can track through to adulthood [9–11], supporting the establishment of healthy dietary habits in childhood has the potential to reduce the burden of both current and future diet-related disease [12, 13].

As schools provide almost universal access to children [14], during which time they consume almost 40 % of their daily energy intake [15], they have been recommended as a key setting for population-based nutrition initiatives [16]. Evidence from systematic reviews suggests that school food and beverage nutrition policies and guidelines have been effective in improving the food environment of schools and the dietary intake of students [17, 18]. As a result, the World Health Organization has recommended that schools implement nutrition policies to control the types of foods and beverages available to students [19]. Accordingly, school healthy eating policies and guidelines have been implemented by various jurisdictions including Canada [20], the United States [21], New Zealand [22], and Australia [23]. For example in Canada the Ontario government’s nutrition standards for schools, which extends to all foods and beverages sold in schools, requires that they ‘sell most’ (at least 80 %) of foods and beverages that are the healthiest options, ‘sell less’ (no more than 20 %) of less healthier options and are not permitted to sell foods or beverages that contain few or no essential nutrients and/or high amounts of fat, sugar, and/or sodium [20]. Similarly, New Zealand schools are encouraged to develop school canteen menus which are mostly made up of ‘every day’ foods and beverages, to not let ‘sometimes’ foods and beverages dominate the menu and that occasional foods and beverages not be sold at all [24].

Although such policies exist, their implementation by schools is less than optimal. For example, results of the 2012 School Health Policies and Practices Study (SHPPS) in the United States found that 57.3 % of secondary schools did not adhere to recommended nutrition standards by selling energy dense nutrient poor foods, including chocolate, pastries, salty snacks and sweetened drinks [25]. Similarly a 2007 study of New Zealand schools found poor adherence to healthy nutrition guidelines where 52 % of school canteen menus did not offer fruit, 24 % did not offer rolls/sandwiches, and only 39 % included water in the menu [26]. Furthermore, a 2012 cross-sectional study of 263 Australian schools found that less than 35 % of schools implemented state-specific healthy canteen policies that restricted the sale of unhealthy foods and beverages [27]. A number of barriers have been reported to impede the implementation of nutrition policies in schools including; insufficient school leadership support [28], a perceived lack of school community support [29], profitability concerns [29], limited nutrition knowledge and food classification skills of food service personnel [30].

To ensure the potential benefits of school healthy eating policies are realised, identification of strategies that are effective in implementing healthy school canteen or nutrition policies is required. A 2010 review by Rabin et al. of the effectiveness of interventions to increase community settings implementation of cancer prevention programs identified just one study which aimed to improve schools’ implementation of healthy eating policies or practices [31]. This multi-component quasi-experimental study was conducted in four matched schools over 4 years and included: training; resources; and financial and in-school advice to support schools’ implementation of healthy food service guidelines [32]. The trial found no significant difference between the intervention and control groups in the fat or sodium content of school cafeteria lunches at follow up.

Given the limited evidence base regarding strategies to increase school implementation of healthy eating policies, further research identifying such strategies that are effective in overcoming schools’ barriers to implementation of nutrition policies that can reach geographically diverse schools in a timely and cost-effective manner is required [33]. In this context, we undertook a study to assess the effectiveness of a theoretically designed multi-strategy intervention in increasing the implementation of a healthy canteen policy in Australian primary schools.

## Methods

### Design and setting

A group randomised controlled trial was conducted in government and Catholic schools located in the Hunter New England (HNE) Local Health District in New South Wales (NSW), Australia. The HNE region covers a large non-metropolitan area (more than 130 000 km^2^); with a demographically and socioeconomically diverse population of children aged 5 to 12 years [34]. This trial was prospectively registered with the Australian New Zealand Clinical Trials Registry (ACTRN12614001148662) on the 30th October 2014.

#### Policy context

In 2005, the NSW state government introduced a healthy school canteen policy (“Fresh Tastes @ School”) [23], mandatory for implementation by state schools and strongly encouraged for use in Catholic schools. Utilising a ‘traffic light’ food classification system, the policy classifies foods and beverages sold in school canteens (whether that be pre-packaged foods or those made on site by canteen staff) as either ‘red’, ‘amber’ or ‘green’ based on their nutritional content (See Tables 1 and 2 below). For all foods sold in the canteen at recess and lunch the policy requires schools to remove all red foods from regular sale and to fill the menu (that is more than 50 %) [35] with green foods and to not let amber foods dominate the menu. Furthermore, in 2007 a ban was introduced on all sugar-sweetened drinks (>300 kJ and/or have >100 mg of sodium/serve), prohibiting them from being sold in schools. Whilst the policy is mandatory in state schools to date there has been no monitoring of implementation and as such no consequences for schools that fail to adhere.

**Table 1:** Classification and examples of Red, Amber and Green items based on “Fresh Tastes @ School”

Red foods	Amber foods	Green foods
‘Red’ foods are nutrient poor, high-energy foods such as confectionary, deep fried foods and chocolate coated or premium ice creams.	‘Amber’ foods are considered to have some nutritional value however if consumed in large amounts can contribute to excess energy intake such as full fat dairy products, processed meats, some snack food bars and biscuits, some savoury snack foods, some muffins and cakes, some ice creams and dairy desserts.	‘Green’ foods are considered to provide good sources of nutrients such as fruit, vegetables, reduced fat dairy products, lean meat, fish and poultry and bottled water.

**Table 2:** The occasional food criteria for determining if a food is red [23]

Hot food assessed per 100 g	Nutrient criteria per 100 g			
Food category	Energy (kJ)		Saturated fat (g)	Sodium (mg)
Savoury pastries, pasta, pizzas, oven baked potato products, spring rolls, fried rice and noodles	>1000 kJ		>5 g	>400 mg
Crumbed & coated foods(e.g., patties, chicken products, frankfurters)	>1000 kJ		>5 g	>700 mg
Snack food and drinks assessed per serve	Nutrient criteria per serve (as sold in canteen)			
Food category	Energy (kJ)	Saturated fat (g)	Sodium (mg)	Fibre (g)
Snack food bars, sweet biscuits	>600 kJ	>3 g		<1.0 g
Savoury snack foods, biscuits	>600 kJ	>3 g	>200 mg	
Ice-creams, milk based ice confections	>600 kJ	>3 g		
Cakes, muffins, sweet pastries	>900 kJ	>3 g		<1.5 g

If the item has more than the number specified in the energy, saturated fat or sodium column, or less than the number in the fibre column, it is a red food

### Participants

Government and Catholic primary schools (children 5 to 12 years of age) in the HNE region with an operational canteen (*n* = 315) served as the sampling frame for the study. Government schools are run by a state government whilst the Catholic schools are run by a diocese-based educational institution. All school systems must follow the same educational curriculum. Schools were ineligible to participate if they; were an independent school, had secondary students (including central schools i.e. enrolling students from Kindergarten to Grade 12), exclusively catered for children requiring specialist care, didn’t have a canteen that operated at least once per week, if they were participating in another canteen intervention study or if they were identified by the NSW government as a high performing health promoting school in terms of implementing nutrition (including canteens) and physical activity policies and practices [36].

### Randomisation, recruitment and allocation

Prior to baseline data collection, schools were randomly allocated in a 1:1 ratio to either an intervention or control group by an independent investigator using a computerised random number function in Microsoft Excel. Group allocation was concealed from staff involved in school recruitment. Such staff contacted school administrators and asked for a copy of the school’s menu to be emailed or faxed to the project team. Schools were not blind to group allocation. Dietitians conducting menu assessments at baseline and follow-up were blind to group allocation.

### Multi-component implementation intervention

The study utilised the Theoretical Domains Framework (TDF) [37] to identify the potential behavioural determinants of implementation of the Fresh Tastes @ School policy as a guide to the selection of implementation intervention strategies. The TDF is an integrative framework of organisational change theory that draws on 33 theories relevant to improving implementation across disciplines. The TDF is comprised of 14 domains and 84 theoretical constructs that allow implementation scientists to assess practitioners’ barriers and enablers to policy implementation, and help inform the design of appropriately targeted interventions. The framework has been widely used in the development of effective clinical practice change interventions [38]. The framework was applied and associated intervention development procedures were used to design the multi-component implementation strategy to improve primary schools’ implementation of the policy. Specifically, implementation of the framework involved the following steps i. Literature reviews of previous nutrition implementation interventions in schools, ii. surveys with canteen managers in the study region using a modified TDF questionnaire [39] and iii. discussions with health promotion practitioners experienced in working with school canteens were undertaken to identify possible barriers and enablers for policy implementation. Utilising such information, the identified barriers were mapped to TDF constructs, and implementation strategies recommended by the TDF to address identified barriers were then selected using a process described by Michie et al. [40]. Delivered over a 9-month period (three school terms October 2014- June 2015) the implementation intervention included:

1. Executive support- School principals were telephoned to inform them of the training and resources available to their school canteen and asked to demonstrate their support for implementation of the Fresh Tastes @ School policy by encouraging the canteen manager and a parent representative to attend canteen manager training and for receipt of ongoing support.
2. Canteen manager/parent training- A 1 day (5 h) group training workshop was offered to canteen managers and parent representatives providing education and skill development in the Fresh Tastes @ School policy, label reading, canteen stock and financial management, pricing and promotion, and change management. Dietitians, experienced in delivering training to canteen managers, conducted the training. The workshop provided opportunities for canteen managers to participate in consensus processes through the development of a canteen action plan identifying how they would implement Fresh Tastes @ School in their school. If a school canteen manager was unable to attend the workshop, they were telephoned and offered a 30–45 min-teleconference call or a face-to-face meeting with a dietitian to discuss workshop content and resources.
3. Tools and resources- Printed instructional materials, sample policies/menus, planning templates, pricing guides, product lists of policy compliant menu items, supplier contacts and menu assessment feedback were provided to all school canteen managers during the workshop or mailed to non-attenders of the workshop. Canteen managers who attended the workshop also received kitchen equipment to the value of AUD$100.
4. On-going support- Following training, canteen managers received two support contacts per school term via text messages. Framed by the TDF these contacts provided targeted advice to overcome common barriers to policy implementation and encouraged canteen managers to review progress against their action plan. Canteen managers who requested additional support were contacted by a project officer after the workshop and provided tailored advice.
5. Performance monitoring and feedback-During the workshop, schools were provided a written feedback report on their previously supplied canteen menu. The feedback report identified the included foods and beverages that were red/banned, amber or green and the proportion of the menu contributed by each category. Red/ banned food items in the report were advised to be removed, with alternatives, where possible, identified. Where amber foods dominated the menu (>50 %), green alternative food items were recommended. The feedback report included a sample ‘compliant’ menu, individually tailored using the schools baseline menu. Canteen managers were asked to send an updated version of the menu for review and a second feedback report was generated.
6. Recognition- Schools with a menu assessed as adhering to the policy (i.e. greater than 50 % green items and no red or banned items) received a congratulatory letter from the research team, and provided a positive feedback article they could include in their school newsletter.

NB: To access intervention materials go to http://www.goodforkids.nsw.gov.au/primary-schools/canteens/.

### Comparison schools

Comparison schools were not offered the multi-strategy intervention described above. However during the trial period, teachers from either intervention or control group schools were able to access NSW Government-run programs directed at supporting school promotion of healthy eating and physical activity generally [41].

### Data collection and measures

#### School characteristics

Data regarding school type (Government, non-Government Catholic), number of students and the postcode of the locality of the school were obtained from the Australian Governments ‘My School’ website [42].

#### Primary trial outcomes

The primary outcomes of the trial were i) the proportion of schools with a canteen menu that did not include red or banned foods and beverages and ii) the proportion of schools where green items make up the majority of the menu defined as more than 50 % of listed menu items [35]. Outcome data were collected at baseline (winter 2014 i.e. May–July 2014) and follow-up (winter 2015 i.e. May–July 2015) via audits of canteen menus faxed or e-mailed to the project team by the school. Trained dietitians, blinded to group allocation, conducted an assessment of the canteen menu using a menu analysis assumptions guide. This method has previously been validated with a cross-sectional study in 38 schools that compared menu analysis using assumptions to an observational audit (the criterion standard) [43]. Observational audits involved 2–3 trained research assistants visiting a school canteen to record the nutritional information from product nutrition panels of all food and beverage items sold in the canteen so that items could be classified according to the Fresh Tastes @ School guidelines. Menu assessment using assumptions was found to have substantial agreement (kappa = 0.68) when compared to direct observation.

#### Delivery of the multi-strategy interventions

Project records were used to assess the fidelity and reach of the intervention in relation to number of schools that were provided each of the implementation intervention strategies.

### Sample size and power

Assuming 80 schools would be assessed as eligible to participate, and a response rate of 70 % would yield a total sample of 56 schools (28 per group). Such a sample would allow the study to detect as significant an absolute change in the primary trial outcomes of approximately 35 with 80 % power and an alpha of 0.05, assuming a control group prevalence of 15 % at follow-up.

### Analyses

All analyses were performed in SAS 9.3 (SAS Institute Inc., Cary, NC). Descriptive statistics were used to describe school characteristics. School postcodes were used to categorise the school’s locality as either ‘rural’ (those schools in outer regional, remote and very remote areas) or ‘urban’ (those in regional cities and inner regional areas) based upon the Australian Standard Geographical Classification (ASGC) (Australian Bureau of Statistics (ABS), 2011). Schools with postcodes ranked in the top 50 % of NSW postcodes based on the Socio-Economic Indexes For Australia (SEIFA) (Australian Bureau of Statistics (ABS), 2011) were categorised as schools in ‘higher socio-economic areas’ while those in the lower 50 % were categorized as schools in ‘lower socio-economic areas’. Menu items were classified and counted from which the percentage of red, amber, green or banned items on each menu could be determined. Descriptive statistics were used to determine the overall percentage of green, amber and red items for the groups. The primary trial outcomes were analysed under an intention-to-treat framework using all available data. Between group differences in the primary outcomes at follow-up were assessed using Fishers exact test and presented as relative risks (with approximate 95 % confidence intervals). In addition a post-hoc analysis was undertaken to determine if implementation of the policy differed by school characteristics. Given only one school was lost to follow-up, sensitivity analyses using imputation to examine the impact of loss to follow-up were not undertaken.

## Results

**Fig. 1:**
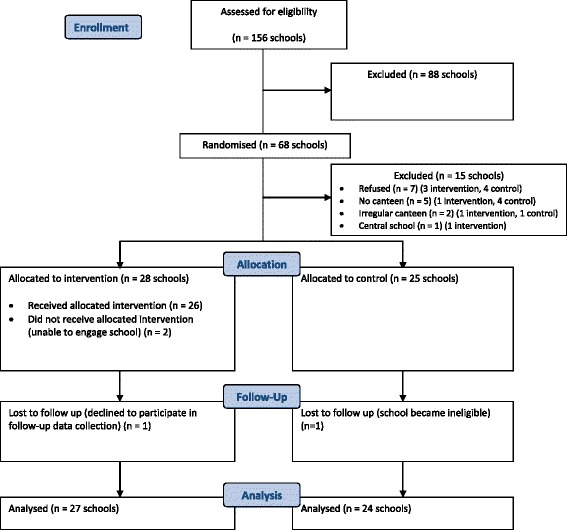
CONSORT flow chart describing progress of participants through the study

Sixty-eight schools were randomised prior to baseline data collection and approached to participate in the study of which 61 schools agreed (89.7 %). However five schools were excluded, as they did not have a canteen and one school was excluded as they were a central school. Of the remaining schools, 55 consented and returned menus (88.7 %) for baseline assessment, two of which were deemed ineligible as they did not have a regular canteen leaving a final baseline sample of 53 schools (28 intervention, 25 control) (Fig. 1 CONSORT). There were no significant differences for schools that consented and participated to those that did not. Furthermore, there were no significant differences between groups in school characteristics or menu composition.

The baseline characteristics of participating schools in intervention and control groups are shown in Table 3. Of the 53 schools, 51 (96 %; 27 intervention and 24 control) provided menus at follow-up.

**Table 3:** Baseline characteristics of participating schools by group

Characteristics	Intervention *N* = 28	Control *N* = 25
School type		
Government Catholic	19 (68 %) 9 (32 %)	16 (64 %) 9 (36 %)
Number of students^†^	232 ± 192	267 ± 209
Urban/Rural region		
Major Cities + Inner Regional	22 (79 %)	23 (92 %)
Outer Regional/Remote Australia	6 (21 %)	2 (8 %)
Socio-economic index		
Lower socio-economic areas	19 (68 %)	18 (72 %)
Higher socio-economic areas	9 (32 %)	7 (28 %)

NB Number of students from one control school is missing

^†^Values reported in mean ± SD

There were no significant differences between groups in school characteristics or menu composition at baseline.

### Primary trial outcomes

As seen in Table 4, intervention schools were significantly more likely than control schools to have a menu without red or banned items (*RR* = 5.78 (1.45–23.05); *p* = 0.002). Similarly, intervention schools were significantly more likely to have at least 50 % of menu items classified as green than control schools (*RR* = 2.03 (1.01–4.08); *p* = 0.03). There were no significant differences in intervention effect based on school characteristics that is school type, geographic or socio-demographic location. The overall percentage green, amber and red menu items for intervention schools at follow-up was 52.0, 45.7 and 2.3 % respectively compared to control schools which had an overall percentage of 47.0 % green, 46.5 % amber and 6.5 % red menu items.

**Table 4:** Relative risk of primary outcome variables at follow-up

	Baseline		Follow-up		Intervention group v control group (95 % CI)	
	Intervention (*N* = 28) n(%)	Control (*N* = 25) n(%)	Intervention (*N* = 27)^a^ n(%)	Control (*N* = 24)^b^ n(%)	Relative risk (95 % CI)	*P*-value
Canteen menu does not contain foods and beverages restricted for sale (red or banned).	5 (17.9)	2 (8.0)	13 (48.2)	2 (8.33)	5.78 (1.45–23.05)	0.002
Healthy canteen items (green) represent >50 % of products listed on the canteen menu.	7 (25.0)	9 (36.0)	16 (59.3)	7 (29.2)	2.03 (1.01–4.08)	0.03

^a^ denotes one school refused to provide follow-up data

^b^ denotes one school canteen closed

### Delivery of the multi-strategy intervention

Table 5 shows the proportion of intervention schools that received each of the implementation strategies. All schools received the resources and kitchen equipment, and most schools (96.4 %) received training, menu feedbacks (92.9 %) and 75 % of canteen managers provided a mobile phone number so that text messages could be distributed.

**Table 5:** Extent of delivery of multi-strategy intervention

Intervention component	Intervention schools (*N* = 28)
Principal engagement	26
Resources (printed and electronic materials)	28
Kitchen equipment	28
Training Workshop	12
Modified training workshop (over phone/ face to face)	14
Action plan follow up contact	21
Menu audit and feedback report	26
Recognition newsletter snippets	14
Number of targeted text messages sent (4 texts per term)	21 provided mobile number for text messages.

## Discussion

This study sought to evaluate the effectiveness of a theoretically designed intervention to facilitate the implementation of a mandatory healthy canteen policy in Australian schools. The findings suggest that a multi-strategy intervention involving training, performance monitoring and feedback, telephone and text messaging support can improve schools’ implementation of a healthy school canteen policy. The study makes a novel contribution to a currently sparse implementation research landscape in the school setting [31] and provides evidence to improve nutrition policy implementation in schools.

The findings contrast with the only previous trial of a strategy to improve school food availability identified in an Agency for Health Care Research and Quality systematic review that found no improvement in food service policy implementation following receipt of training, resources, financial and in-school advice [32]. The effect sizes for the primary trial outcomes in this study (25–42 % relative to comparison schools) are however consistent with trials of other interventions that have sought to enhance implementation of a vegetable and fruit program in schools specifically [44] or other health promotion programs generally [45–47] that have used similar implementation support strategies (13–45 %). Given previously reported evidence that changing the relative availability of healthy food in schools can improve student diet [48, 18], the findings suggest that the provision of implementation support to school canteens has the potential to make a meaningful contribution to improving child nutrition, health and well-being. Despite the success of the intervention in terms of the primary outcome measures, 52 % of schools continued to include red items on their canteen menu. 41 % of schools continued to have menus where the majority of items were not classified as green. Given this, further research to identify strategies that are effective in improving food availability for sale by all schools is warranted to ensure all children gain the intended benefits of healthy school canteen policies.

The use of an implementation theoretical framework to guide the development of the intervention was a strength of the study. Whilst the findings suggest that the intervention enabled schools to overcome barriers to policy implementation, the size of the study sample precluded verification of this hypothesis empirically. Examining the impact of the intervention on the antecedents to school canteen policy implementation for example through mediation analyses would represent particularly useful additional research for researchers, policy makers and practitioners to better understand intervention mechanisms and identify implementation strategies that could be added to enhance effect size, or removed to enhance intervention cost-effectiveness. The lack of psychometrically robust, theoretically informed tools to assess implementation barriers in the school setting is an impediment to such research. Addressing this gap in the scientific literature should be seen a priority to advance the field of implementation science and improve the impact of strategies to implement evidence-based nutrition policies.

The study findings should be considered in the context of the trial methods. The study is strengthened by the trial’s randomised controlled design, the theoretical basis for the implementation intervention, blinded outcome assessment and high study retention at follow-up. However, given schools were sampled from only one region within New South Wales the generalizability of the findings to other school systems, or other jurisdictions is limited. Encouragingly though, at least within the study sample, there appeared little difference in the effect of the implementation strategy according to school characteristics suggesting that the intervention may be similarly effective across a variety of socioeconomic and geographic localities. The trial did also not assess canteen manager’s satisfaction with the intervention. Whilst the high level of reach would suggest that the intervention was acceptable to the canteen managers, the collection of such process data could have informed future implementation interventions.

## Conclusion

Low rates of implementation of school canteen policies in Australia have persisted for more than a decade since policy release, despite government investment in supportive infrastructure. Whilst multi-strategic interventions are often recommended for school-based interventions the cost to government agencies to deliver such interventions at scale is often challenging. The use of telephone and text messaging support employed in this trial enhances the potential scalability of this intervention, thereby providing novel information for public health policy makers and practitioners regarding strategies to facilitate the implementation of nutrition policies and guidelines broadly, and healthy canteen policies specifically.
